# Impact of dog sports participation on quality of life, dog owner relationship, and physical activity in women

**DOI:** 10.3389/fspor.2026.1872198

**Published:** 2026-09-03

**Authors:** Marta Gimunová, Gabriela Kuklová, Michaela Wagner, Emma Benýšková, Viktorie Bulínová, Lucie Lipková, Adam Wagner, Michal Bozděch

**Affiliations:** 1 Department of Physical Activities and Health Sciences, Faculty of Sports Studies, Masaryk University, Brno, Czechia; 2 Department of Sport Performance and Exercise Testing, Faculty of Sports Studies, Masaryk University, Brno, Czechia; 3 Department of Physical Education and Social Sciences, Faculty of Sports Studies, Masaryk University, Brno, Czechia

**Keywords:** dog sports, human-animal interaction, human-dog interaction, pet owners, physical activity

## Abstract

**Introduction:**

Differences in physical activity or perceived quality of life between dog owners and people without a dog were observed previously. However, the differences between dog pet owners and dog owners participating in dog sports are not well analyzed. Therefore, the aim of this study was to assess the differences in quality of life, dog owner relationship, and physical activity in female dog owners participating in dog sports compared with female dog owners with no dog sports history.

**Methods:**

A total of 162 women participated in this study (90 women with regular trainings and competitions in dog sports, 72 women with no trainings or competitions in dog sports). All participants completed an online survey consisting of Monash Dog Owner Relationship Scale, The World Health Organization Quality of Life—Brief, and International Physical Activity Questionnaire—Short Form.

**Results:**

Results show a higher Metabolic Equivalent of Task (MET) moderate physical activity in female dog owners participating in dog sports. A significant correlation between dog trainings (minutes per week) and MET vigorous, MET moderate, and MET total was observed, suggesting a contribution of dog sports participation in higher levels of physical activity. Additionally, in female dog owners participating in dog sports, the quality of life correlated with body mass index (BMI) and dog owner relationship.

**Discussion:**

These findings suggest that dog sports may support an active lifestyle among women, and that among women actively involved in dog sports, the perceived quality of life is positively related to the frequency of shared interactions and the overall strength of the dog owner relationship. This aligns with previous evidence that dog-sport competitors are highly driven by intrinsic motivations, such as the enjoyment of shared activities, and strengthening of the human-animal bond. Future research including objective measures of physical activity, and more diverse samples will allow better understanding the health and well-being implications of participation in dog sports.

## 1 Introduction

Owning a dog has a significant impact on a person's life. Contact with pets, especially dogs and cats, has been shown to reduce stress, anxiety, and blood pressure in their owners, thereby lowering the risk of cardiovascular disease (1–3). Dog ownership is also associated with higher levels of physical activity. Studies show that dog owners tend to be more active than people without dogs (4, 5). A previous study by Astuti et al. (2) examined the effects of dog ownership on women's physical and mental health. The study reported lower levels of stress and anxiety, improved mood and motivation, strengthened social bonds, and greater perceived emotional support. However, the owners also faced financial and time demands associated with having a dog, as well as concerns about illness or loss of the pet (2).

Dog sports are a rapidly growing sport domain involving a partnership between a dog and the handler and challenging their physical and cognitive skills. Participation in dog sports could have positive effects on dog handler's and dog's health (6). Dog sports cover various disciplines such as agility, obedience, rally obedience, mushing, and working trials. Motivations for dog sports participation vary from leisure time activities to professional commitment and the competition level ranges from local to international level. Dogs that are actively trained and compete in dog sports disciplines receive more than one hour of daily physical activity (e.g., walks), one hour of vigorous physical conditioning exercise per week and more than three hours of sport-specific training per week (7). These training loads suggest that dog sports can meaningfully increase total weekly activity in handlers. Notably, in young adult female dog handlers, dog sports were observed to be as effective as other sports to induce health benefits (8).

Previous studies show differences in physical activity or perceived quality of life between dog owners and people without a dog (e.g., 4, 5). However, the differences between dog pet owners and dog owners participating in dog sports are not well analyzed. Therefore, the aims of this study were (i) to assess the differences in quality of life, dog owner relationship, and physical activity in female dog owners participating in dog sports compared with female dog owners without a history of dog-sport participation; (ii) to assess the relationship between dog training duration (minutes per week) and total physical activity; (iii) to assess the relationship between quality of life, dog owner relationship, and physical activity in female dog owners participating in dog sports and female dog owners without a history of dog-sport participation.

## 2 Methods

A total of 162 female dog owners participated in this study by completing an online survey via Google forms. From these, 90 women had regular trainings and competitions in dog sports (Dog sports group), 72 women reported no trainings or competitions with their dog (Dog pet group). The study was conducted in accordance with the Declaration of Helsinki, and participants provided their informed consent online before participating in the survey. The study did not require approval from an institutional ethics committee as the survey was conducted anonymously, did not collect any personally identifiable information, and did not involve sensitive personal data or vulnerable populations.

Inclusion criteria consisted of the age 18 years or over, female gender, owning a dog, and ability to complete the entire survey. Exclusion criteria were age under 18 years, male gender, and not completing the entire survey.

### 2.1 Survey sections

The following sections were included in the survey: demographics, Monash Dog Owner Relationship Scale (MDORS), The World Health Organization Quality of Life—Bref (WHOQOL-BREF) and International Physical Activity Questionnaire—Short Form (IPAQ-SF).

#### 2.1.1 Demographics

This section included demographical information about the participants’ age, gender, body height, and body weight, education, information about their dog (breed, and size), participation in dog sports, its discipline, and competitions experience.

#### 2.1.2 Monash Dog owner relationship scale (MDORS)

MDORS is a 28-item questionnaire measuring the owner's perceived relationship with their dog. MDORS is one of the few questionnaires focusing exclusively on a human-animal relationship in a single animal species. This questionnaire includes three subscales: (i) dog owner interactions; (ii) perceived emotional closeness; (iii) and perceived costs (9). Higher scores in dog owner interactions subscale mean higher level of interactions (e.g., playing games, hugging, kissing or giving food treats), higher scores in perceived emotional closeness subscale mean higher emotional closeness (e.g., constant companionship with the dog, dog is the reason to get up in the morning, or dog helping to get through tough times), higher scores in perceived costs subscale mean lower perceived cost for the owner (e.g., changing plans because of the dog, cost of money, or feeling that looking after the dog is a chore) (10).

#### 2.1.3 The world health organization quality of life—bref (WHOQOL-BREF)

The quality of life in this study was examined using a questionnaire developed by the World Health Organization. WHOQOL-BREF is a shortened version of the WHOQOL-100 questionnaire. It consists of 26 questions, two of which are individual items assessing the respondent's overall subjective quality of life and health on a Likert scale. The remaining 24 questions are divided into four domains evaluating: (i) physical health (e.g., pain, energy, and sleep); (ii) psychological domain (e.g., positive feeling, self-esteem, and body image); (iii) social relationships (e.g., personal relationships, and social support); (iv) and environmental domain (e.g., safety, access to transport, and financial resources). Each domain is transformed to a 0–100 scale and averaged for a global score. A higher score indicated higher quality of life (11).

#### 2.1.4 International physical activity questionnaire—short form (IPAQ—SF)

This questionnaire provides an overview of the respondents’ physical activity during the past 7 days. The questionnaire asks about the intensity, duration, and frequency of activities. The IPAQ-SF focuses on four areas: vigorous-intensity activity, moderate-intensity activity, walking, and sedentary activity. Minutes of activity at different intensities are converted into Metabolic Equivalent of Task (MET), where 1 MET corresponds to the energy expenditure at rest. Walking requires more energy, so in the IPAQ-SF, the time spent walking is multiplied by 3.3, moderate-intensity activity by 4, and vigorous-intensity activity by 8. The result estimates the total amount of physical activity in METs per week (12).

### 2.2 Statistical analysis

Due to violation of normality, a non-parametric approach was applied. Differences in quality of life, dog owner relationship, and physical activity between dog sports and dog pet groups were analyzed using Mann–Whitney U test. To assess the relationship between dog trainings duration (minutes per week) and total physical activity, Spearman's correlation was used. Spearman's correlation was used to assess the relationship between quality of life and dog owner relationship, and physical activity in female dog owners participating in dog sports and female dog owners with no dog sports history. The level of significance was set at *α* = 0.05. The statistical analysis was performed using JASP (v.019.3.0). Effect size was analyzed using Cohen's d and interpreted as small (≥0.20), medium (≥0.50), and large (≥0.80) effect (13).

*A priori* power analysis was performed in G*Power (version 3.1.9.4) for the Wilcoxon–Mann–Whitney test for two independent groups to calculate sample size. The calculation was based on data published by Karvinen and Rhodes (18), specifically the variable Total Physical Activity with a Dog. Assuming an effect size of *d* = 0.408 and significance level of *α* = 0.05, the estimated sample size was *n* = 158 (i.e., 79 participants for each group). The current sample size in this study (*n* = 72 and *n* = 90 for dog pet and dog sports groups, respectively) corresponded to a statistical power of 80.7%.

## 3 Results

### 3.1 Participants’ characteristics

In Table 1, characteristics of participants from dog sports group (*n* = 90), and dog pet group (*n* = 72) are shown. Statistically significant difference was observed in the age of the participants from dog sports and dog pet groups. No statistically significant difference in body weight, body height, and body mass index (BMI) was observed.

**Table 1:** Participants’ characteristics.

						95% CI for ES	
Variable	Group	Mean	SD	*p*	ES	Lower	Upper
Age (years)	dog pet group	39.61	10.88	0.048^a^	0.181	0.003	0.347
	dog sports group	36.44	10.65				
Body weight (kg)	dog pet group	69.07	13.24	0.706	−0.035	−0.211	0.144
	dog sports group	68.44	10.25				
Body height (cm)	dog pet group	168.58	6.84	0.874	0.015	−0.163	0.192
	dog sports group	167.93	6.67				
BMI (kg/m^2^)	dog pet group	24.28	4.11	0.734	−0.031	−0.208	0.147
	dog sports group	24.25	3.28				

BMI, body mass index.

a Significant at 0.05.

Maximal education level of participants was primary school level (1.1%, and 1.4% in dog sports and dog pet groups, respectively), secondary school level (40.0%, and 45.9% in dog sports and dog pet groups, respectively), and university level (58.9%, and 52.8% in dog sports and dog pet groups, respectively).

Most of the participants owned a purebred dog (75.6% and 73.6% in dog sports and dog pet groups, respectively). The size of the dog ranged between mini to giant: mini (< 2 kg; 0%, and 2.8% in dog sports and dog pet groups, respectively), small (2–10 kg; 14.4%, and 16.7% in dog sports and dog pet groups, respectively), medium (11–25 kg; 67.8%, and 52.8% in dog sports and dog pet groups, respectively), large (26–50 kg; 16.7%, and 25.0% in dog sports and dog pet groups, respectively), giant (>50 kg; 1.1%, and 2.8% in dog sports and dog pet groups, respectively).

Most of the participants from the dog sports group reported training in agility (*n* = 36; 40.0%), obedience, obedience trial or rally obedience (*n* = 31; 34.4%), canicross (*n* = 26; 28.9%), and heelwork to music (*n* = 11; 12.2%). In our dog sports sample, 43.3% (*n* = 39) of participants reported training in one dog sport discipline; 56.7% (*n* = 51) of participants combine two to five disciplines (Supplementary Material). Most of the participants find their dog sport physically demanding (*n* = 72; 80%).

### 3.2 Differences in quality of life, dog owner relationship, and physical activity between dog sports and dog pet groups

#### 3.2.1 Quality of life

**Figure 1:**
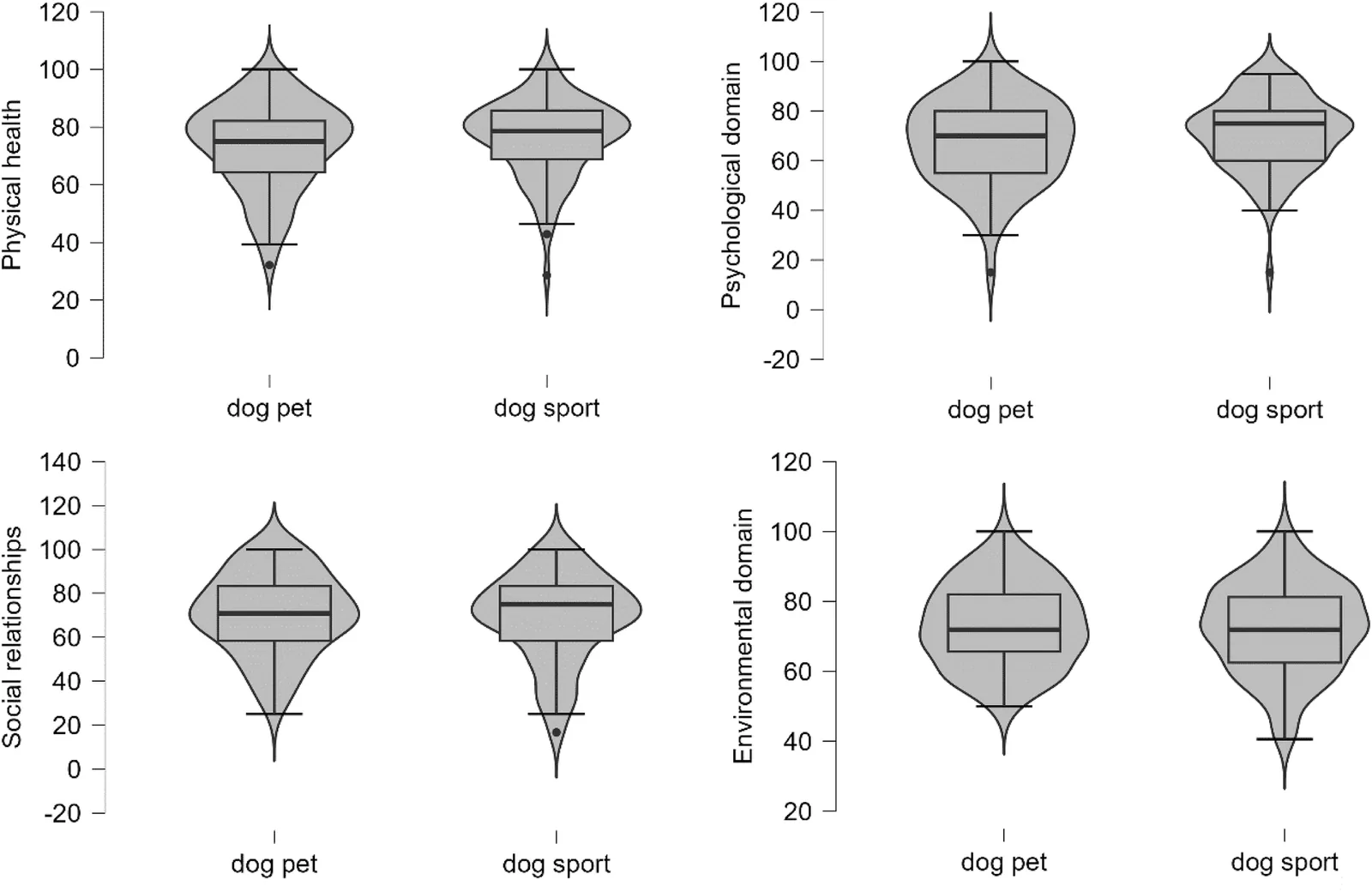
Violin plots showing quality of life (based on WHOQOL-BREF) differences between dog sports group and dog pet group.

No statistically significant difference in subjective quality of life, health, physical health, psychological domain, social relationships, and environmental domain was observed between the dog sports group and dog pet group (Table 2, Figure 1).

**Table 2:** Quality of life (based on WHOQOL-BREF) differences between dog sports group and dog pet group.

						95% CI for ES	
Variable	Group	Mean	SD	*p*	ES	Lower	Upper
Quality of life	dog pet group	4.29	0.68	0.291	0.091	−0.260	0.092
	dog sports group	4.41	0.62				
Health	dog pet group	3.76	0.90	0.061	0.091	−0.323	0.024
	dog sports group	4.02	0.79				
Physical health	dog pet group	73.36	15.26	0.252	−0.105	−0.277	0.074
	dog sports group	76.27	13.18				
Psychological domain	dog pet group	67.01	16.99	0.151	−0.131	−0.302	0.048
	dog sports group	70.94	14.59				
Social relationships	dog pet group	70.49	18.55	0.779	0.026	−0.153	0.202
	dog sports group	68.70	19.92				
Environmental domain	dog pet group	73.26	11.95	0.805	0.023	−0.156	0.199
	dog sports group	72.29	12.91				

#### 3.2.2 Dog owner relationship

**Figure 2:**
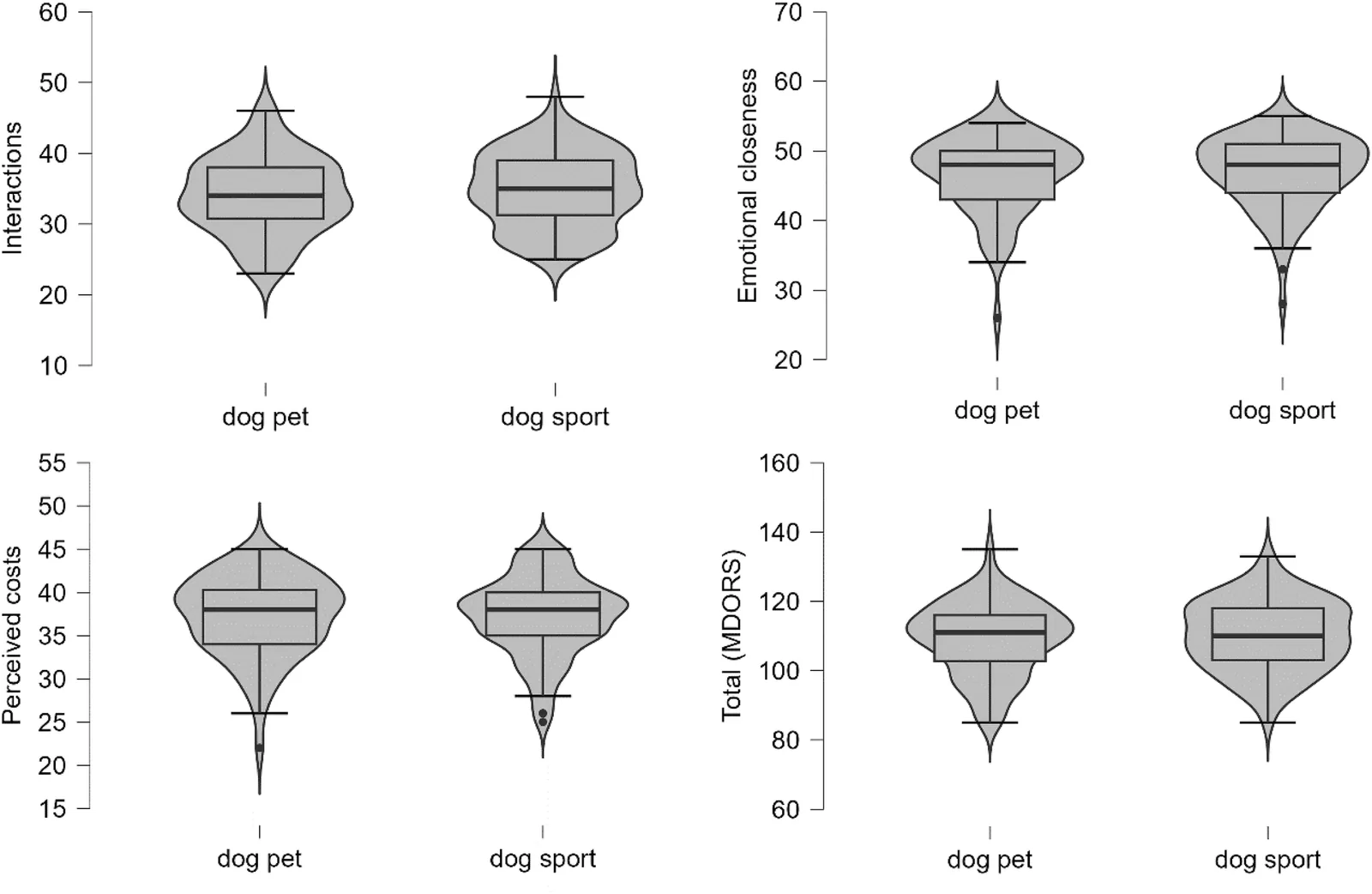
Violin plots showing dog owner relationship (based on MDORS) differences between dog sports group and dog pet group.

No statistically significant difference in the total score and the three subscales of MDORS (dog owner interactions; perceived emotional closeness; and perceived costs) between the dog sports group and dog pet group was observed (Table 3, Figure 2).

**Table 3:** Dog owner relationship (based on MDORS) differences between dog sports group and dog pet group.

						95% CI for ES	
Variable	Group	Mean	SD	*p*	ES	Lower	Upper
Interactions	dog pet group	34.06	5.43	0.322	−0.091	−0.264	0.088
	dog sports group	34.89	5.30				
Emotional closeness	dog pet group	46.54	5.44	0.701	−0.035	−0.211	0.143
	dog sports group	46.93	5.17				
Perceived costs	dog pet group	37.25	4.62	0.779	0.026	−0.152	0.202
	dog sports group	37.14	4.54				
Total	dog pet group	109.11	10.48	0.650	−0.042	−0.218	0.137
	dog sports group	110.14	10.09				

#### 3.2.3 Physical activity

**Figure 3:**
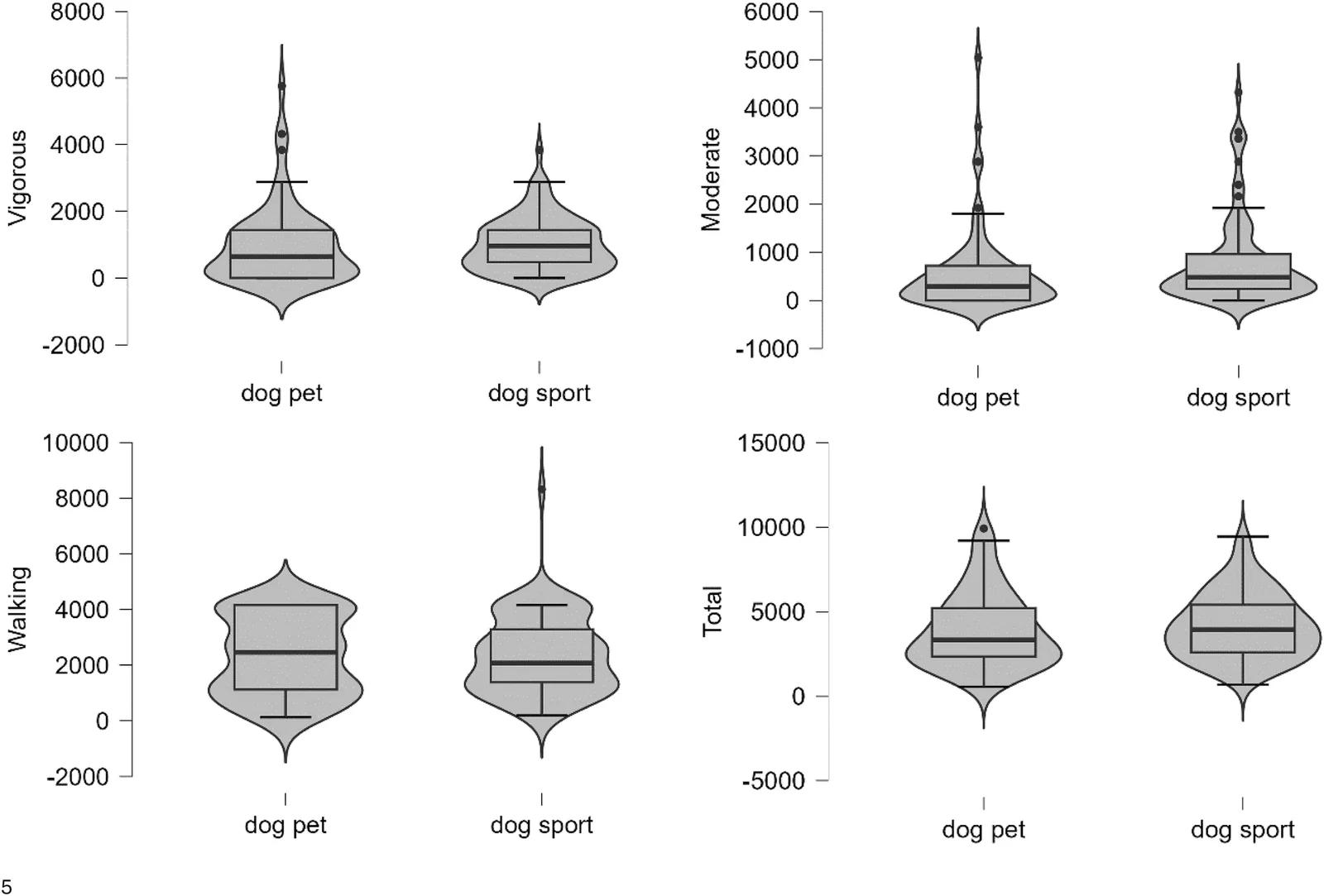
Violin plots showing physical activity in MET (based on IPAQ-SF) differences between dog sports group and dog pet group.

A significantly higher amount of MET moderate was observed in dog sports group compared to dog pet group (*p* = 0.016). No statistically significant differences in MET vigorous, MET walking, and MET total were observed (Table 4, Figure 3).

**Table 4:** Physical activity (based on IPAQ-SF) differences between dog sports group and dog pet group.

		MET		Minutes per week				95% CI for ES	
Variable	Group	Mean	SD	Mean	SD	*p*	ES	Lower	Upper
Vigorous	dog pet group	991.56	1166.38	123.95	145.80	0.218	−0.112	−0.284	0.067
	dog sports group	1004.44	808.42	125.56	101.05				
Moderate	dog pet group	577.22	880.90	144.31	220.23	0.016^a^	−0.220	−0.383	−0.044
	dog sports group	792.62	908.88	198.16	227.22				
Walking	dog pet group	2318.94	1409.49	702.71	427.12	0.905	−0.011	−0.188	0.167
	dog sports group	2361.70	1383.42	715.67	419.22				
Total	dog pet group	3887.72	2221.19	970.96	793.14	0.203	−0.117	−0.288	0.062
	dog sports group	4158.77	1927.93	1039.38	747.49				

MET, metabolic equivalent of task.

a Significant at 0.05.

### 3.3 The relationship between dog trainings duration and physical activity

Additionally, a significant correlation between dog trainings (minutes per week) and MET vigorous (Spearman's rho=0.21, *p* = 0.043), MET moderate (Spearman's rho=0.29, *p* = 0.005), MET total (Spearman's rho=0.27, *p* = 0.010) was observed in the dog sports group. Correlation between dog training (minutes per week) and MET walking did not reach a statistical significance (Spearman's rho=0.09, *p* = 0.424).

### 3.4 Associations between quality of life, MDORS, and physical activity

In dog sports group, a significant correlation between overall quality of life and BMI (Spearman's rho=-0.22, *p* = 0.037), MDORS interactions (Spearman's rho=0.24, *p* = 0.023), total score of MDORS (Spearman's rho=0.21, *p* = 0.044), and MET total (Spearman's rho=0.22, *p* = 0.036) was observed. Significant correlation was observed also between overall health and BMI (Spearman's rho=-0.28, *p* = 0.008), and MET walking (Spearman's rho=0.22, *p* = 0.034). Physical health domain correlated with BMI (Spearman's rho=−0.33, *p* = 0.001). In the psychological domain, a significant correlation was observed with age (Spearman's rho=0.32, *p* = 0.002), BMI (Spearman's rho=−0.29, *p* = 0.005), and MET total (Spearman's rho=0.24, *p* = 0.024). In the environmental domain, a significant correlation was observed with MET total (Spearman's rho=0.21, *p* = 0.043).

In dog pet group, a significant correlation between physical health domain and MET moderate (Spearman’s rho=0.26, *p* = 0.029), psychological domain and age (Spearman’s rho=0.23, *p* = 0.049), and environmental domain and MET vigorous (Spearman’s rho=0.25, *p* = 0.036) was observed (Table 5).

**Table 5:** Spearman's correlation between quality of life (based on WHOQOL-BREF) and age, BMI, dog owner relationship (based on MDORS), and physical activity (based on IPAQ-SF).

		Quality of life		Health		Physical health		Psychological domain		Social relationships		Environmental domain	
Variable	Group	Dog sports	Dog pet	Dog sports	Dog pet	Dog sports	Dog pet	Dog sports	Dog pet	Dog sports	Dog pet	Dog sports	Dog pet
	Age (years)	0.12	−0.02	0.13	0.12	0.19	0.12	0.32^a^	0.23^a^	0.07	0.02	0.02	−0.06
	BMI	−0.22^a^	−0.03	−0.28^a^	−0.14	−0.33^a^	−0.09	−0.29^a^	0.00	0.01	−0.11	−0.14	0.02
MDORS	Interactions	0.24^a^	0.08	0.14	0.05	0.04	−0.16	0.17	−0.15	0.02	−0.16	0.15	0.17
	Emotional closeness	0.11	0.21	0.02	0.13	−0.12	−0.06	0.08	0.09	−0.04	0.00	0.02	0.09
	Perceived costs	0.13	0.03	0.04	0.10	0.14	0.05	0.17	−0.01	0.03	−0.08	0.09	−0.07
	Total	0.21^a^	0.14	0.09	0.10	0.01	−0.07	0.15	−0.02	−0.04	−0.09	0.11	0.11
IPAQ-SF	MET vigorous	−0.06	0.19	−0.03	0.13	0.02	0.15	0.04	0.10	0.02	0.09	0.09	0.25^a^
	MET moderate	0.19	0.01	−0.06	0.06	−0.12	0.26^a^	0.08	0.08	0.06	0.04	−0.11	0.14
	MET walking	0.15	−0.11	0.22^a^	−0.17	0.09	−0.17	0.17	−0.17	0.07	−0.18	0.19	−0.21
	MET total	0.22^a^	0.03	0.16	−0.00	0.12	0.00	0.24^a^	−0.07	0.15	−0.14	0.21^a^	−0.02

BMI, body mass index; MET, metabolic equivalent of task; MDORS, monash dog owner relationship scale; IPAQ-SF, international physical activity questionnaire–short form.

a Significant at 0.05.

## 4 Discussion

The aim of this study was to compare the quality of life, dog owner relationship and physical activity in female dog owners participating in dog sports compared with female dog owners with no dog sports history. The results show a higher MET moderate physical activity in female dog owners participating in dog sports. A significant correlation between dog trainings (minutes per week) and MET vigorous, MET moderate, and MET total was observed, suggesting a contribution of dog sports participation in higher levels of physical activity. Additionally, this study aimed to assess the relationship between quality of life, dog owner relationship, and physical activity. In both groups, the quality of life was correlated with age and physical activity. Additionally, in female dog owners participating in dog sports, the quality of life correlated with BMI and dog owner relationship.

The present study showed higher moderate-intensity physical activity in women participating in dog sports compared with women in the dog pet group, whereas vigorous-intensity physical activity, walking, and total physical activity did not differ significantly between groups. This pattern suggests that dog sports participation may be associated specifically with moderate-intensity activity rather than with a generally higher physical activity profile. Since all participants were dog owners, the lack of difference in walking MET is not surprising and likely reflects regular dog walking in both groups. Previous literature has consistently shown that dog ownership and dog walking are associated with higher walking, and leisure-time physical activity compared with non-dog owners, although not all dog owners achieve recommended physical activity levels (4, 14, 15). The present findings indicate that, within dog owners, participation in dog sports may add a further structured component of moderate-intensity activity.

This interpretation is supported by the positive associations between weekly dog training duration and vigorous-intensity, moderate-intensity, and total physical activity in the dog sports group. Although the observed correlations were small, they indicate that greater engagement in dog sports was linked to higher physical activity levels. In contrast, training duration was not associated with walking, suggesting that dog sports contribute mainly through sport-specific training rather than routine dog walking. As evidence directly addressing dog sports and human physical activity remains limited (8), the present study contributes to a still underexplored area. Nevertheless, the findings should be interpreted cautiously, as the physical demands of dog sports differ across disciplines (7) and self-reported questionnaires may not fully reflect the specific nature of this type of activity.

Although women participating in dog sports did not report a significantly higher overall quality of life than women in the dog pet group, several within-group associations observed in the dog sports group provide a clearer picture. The absence of between-group differences suggests that participation in dog sports alone is not sufficient to differentiate subjective quality of life. This is consistent with broader literature demonstrating that the association between pet ownership and human mental health or well-being is complex and not uniformly positive across studies (16). One possible explanation is that dog ownership itself already provides psychosocial and emotional benefits across both groups (2), thereby reducing the likelihood of detecting additional quality of life differences caused specifically by dog sports participation.

In the dog sports group, overall quality of life was positively associated with MDORS interactions and the total MDORS score. This indicates that among women actively involved in dog sports, perceived quality of life is positively related to the frequency of shared interactions and the overall strength of the dog owner relationship. This aligns with evidence that dog-sport competitors are highly driven by intrinsic motivations, particularly the enjoyment of shared activities, the team aspect of training, and the strengthening of the human-animal bond (17). Moreover, research comparing agility trainers to non-competitors confirms that sports participants exhibit significantly higher competence and social motives for physical activity with their dogs (18). Previous research supports this perspective, showing that stronger human-animal bonds and relational aspects of dog ownership, such as engaging in shared activities and lower perceived costs, are significant determinants of owner satisfaction and well-being (19).

Furthermore, overall quality of life in the dog sports group was positively associated with total physical activity and negatively associated with BMI. These findings are in line with wider literature showing that higher levels of physical activity are robustly associated with improved quality of life and well-being (20), whereas higher BMI is often related to poorer health-related quality of life, particularly in physical domains (21). The finding that physical activity is frequently cited as a primary motivator for engaging in dog sports (17), and that dog sport participants accumulate significantly more moderate-to-vigorous physical activity specifically with their dogs compared to non-competitors (18), further supports that in these women, quality of life reflects broader lifestyle-related factors, including overall physical activity, body composition, and the perceived relationship with the dog, rather than just the act of participating in dog sports.

Because the study was cross-sectional, these associations should be interpreted cautiously and cannot be considered causal. It remains unclear whether better quality of life promotes stronger engagement with the dog and a more active lifestyle, or whether these factors contribute to better perceived quality of life.

There are several limitations of this study. We recognize that this design does not allow for causal inference; thus, results should be interpreted cautiously and may reflect reverse causality or self-selection in dog sports. The physical activity was measured using the IPAQ-SF, which is subject to recall and social desirability bias and assesses physical activity only in the preceding 7 days. Furthermore, the online recruitment may have selected a sample of more engaged and health-conscious dog owners and the study recruited only women, so caution is warranted when generalizing the findings to other groups, such as men or mixed-gender groups.

## 5 Conclusions

This study aimed to analyze quality of life, the dog owner relationship, and physical activity between female dog owners engaged in dog sports and those owning pet dogs. The findings indicate that participation in dog sports is associated with significantly higher levels of moderate physical activity, and that greater time spent on dog training correlates with higher vigorous, moderate, and total physical activity. No significant differences were observed between the groups in overall quality of life or dog-owner relationship. However, within the dog sports group, higher quality of life was associated with better dog owner interactions, greater total PA, and lower BMI. These findings suggest that dog sports may support an active lifestyle among women, and that among women actively involved in dog sports, the perceived quality of life is positively related to the frequency of shared interactions and the overall strength of the dog owner relationship. Future research should employ objective measures of physical activity and analyze the intensity-specific contributions of different dog sports to better understand the health and well-being implications of participation in dog sports.

## Data Availability

The raw data supporting the conclusions of this article will be made available by the authors, without undue reservation.
